# Chemical Profiles and Biological Activities of Essential Oil from *Serissa japonica*

**DOI:** 10.3390/molecules30122485

**Published:** 2025-06-06

**Authors:** Ty Viet Pham, Thien-Y Vu, Hien Minh Nguyen

**Affiliations:** 1 Faculty of Chemistry, University of Education, Hue University, 34 Le Loi, Hue City 530000, Vietnam; phamvietty@hueuni.edu.vn; 2 Faculty of Pharmacy, Ton Duc Thang University, Ho Chi Minh City 700000, Vietnam; vuthieny@tdtu.edu.vn; 3 Research Group in Pharmaceutical and Biomedical Sciences, Faculty of Pharmacy, Ton Duc Thang University, Ho Chi Minh City 700000, Vietnam

**Keywords:** *Serissa japonica*, essential oils, (*E*)-nerolidol, antioxidant, anti-inflammatory, anti-glucosidase, in silico study

## Abstract

This study was the first to analyze the chemical compositions and bioactivities of *Serissa japonica* leaf oil. The oil, obtained via hydro-distillation with a 0.1% yield, contained 64 compounds, predominantly non-terpenic compounds (39.0%), oxygenated sesquiterpenes (31.4%), and oxygenated monoterpenes (25.6%). Major constituents included 1,8-cineole, (*E*)-nerolidol, and *iso*-longifolol. The oil showed good antioxidant activity (IC_50_ ≈ 62.79 ± 0.77 µg/mL for DPPH and 57.82 ± 1.12 µg/mL for ABTS) and a good anti-tyrosinase effect (IC_50_ ≈ 195.6 ± 3.82 µg/mL). The trend was similar to anti-inflammatory activity, with an IC_50_ value of 63.03 ± 3.22, for NO inhibition without cytotoxicity at 100 µg/mL. The bovine serum albumin (BSA) blocking assay demonstrated an IC_50_ value of 59.31 ± 0.71 µg/mL, indicating a good interaction regarding enzyme inhibition. Moreover, the computational modeling of the possible association between tyrosinase and cyclooxygenase-2 highlighted their antioxidant and anti-inflammatory properties. The results pointed out the usefulness of *S. japonica* essential oil as a natural candidate for managing oxidative stress and inflammation.

## 1. Introduction

Essential oils (EOs) are a rich source of plant-derived compounds known for their antioxidant, anti-inflammatory, and anti-glucosidase properties by reducing oxidative stress and modulating immune response [1,2]. The antioxidants in EOs help alleviate oxidative stress by neutralizing harmful radicals, thus safeguarding cells and supporting overall well-being [3]. The secondary metabolites found in EOs, such as phenolic compounds, monoterpenes, and sesquiterpenes, impact the mechanisms of inflammation and the immune response. As a result, they exhibit significant anti-inflammatory activity [4,5]. Chronic inflammation and oxidative stress are interconnected pathological processes that contribute to cancer development and progression. The pathophysiology of several age-related degenerative diseases, including diabetes, cancer, and heart failure, is closely linked to oxidative DNA damage [6]. Oxidative stress, hyperglycemia, and the emergence of diabetic complications are strongly correlated [7]. Inflammation and oxidative stress are both causes and effects of diabetic kidney diseases, so they could be regarded as reciprocal causes of the illness. Moreover, EOs have attracted attention for their potential anti-glucosidase activity, which may aid in managing type 2 diabetes. The regulation of blood glucose levels relies on *α*-glucosidase, an enzyme that plays an essential role in carbohydrate metabolism [8]. By blocking this enzyme, EOs could assist in slowing carbohydrate breakdown [9].

Plants in the Rubiaceae family have shown potential health benefits. They contain compounds that may help lower blood pressure, reduce cholesterol, manage blood sugar, and provide anti-inflammatory and antioxidant effects [10]. This family is edible and rich in bioactive compounds that affect multiple metabolic pathways. The structural diversity of terpenoids, anthraquinones, iridoids, and indole alkaloids adds to their worth as nutraceuticals, offering a range of health-promoting effects [11]. The phytochemical investigations of the *Serissa* genus have obtained several metabolites, including triterpenes, steroids, and lignans. The leaves, stems, and roots are commonly processed into powders, brewed as teas, or boiled to create extracts. The *S. japonica* is a flowering plant species in the Rubiaceae family. This species is native to subtropical forests and moist grasslands in Southeast Asia, ranging from India and China to Japan. The plant grows 2–4 feet tall and slightly wider, with rigid branches covered in small, glossy leaves and numerous white flowers [12]. Recent research has revealed that *S. japonica* contained a distinct array of lignan compounds, comprising furofuran-, tetrahydrofuran-, and arylnaphthalene-type lignans [13].

Despite the broad range of therapeutic benefits associated with this family and genus, there is a lack of scientific data specifically supporting *S. japonica* EO. For the first time, chemical compositions and biological activities such as antioxidants (DPPH and ABTS), anti-tyrosinase, anti-inflammatory (inhibition of nitric oxide (NO) production and bovine serum albumin (BSA) denaturation), and anti-α-glucosidase activities were evaluated. Molecular docking analysis was performed to elucidate the interactions between the predominant bioactive compounds and their target enzymes.

## 2. Results

### 2.1. GC-MS Profiles

**Figure 1:**
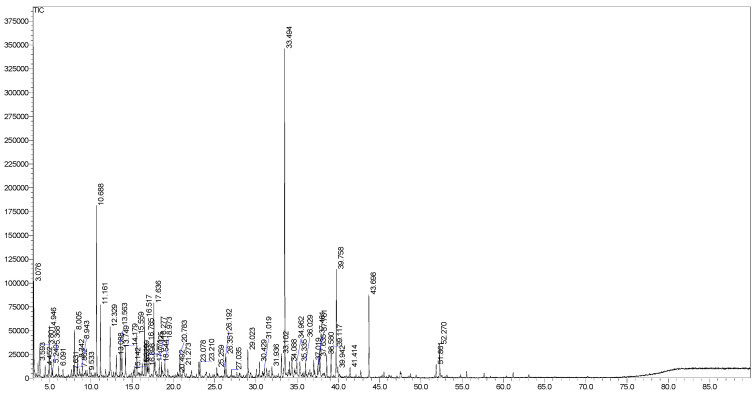
GC chromatogram of *Serissa japonica* leaf EO.

The EO from *S. japonica* leaves was obtained as a yellow liquid with a yield of 0.1% (*v*/*w*, fresh weight basis), typical of low-yielding species within the Rubiaceae family. GC-MS analysis identified 64 compounds, accounting for 99.4% of the oil’s composition (Figure 1, Table 1). Non-terpenic compounds were the dominant class (39.0%), followed by oxygenated sesquiterpenes (31.4%), oxygenated monoterpenes (25.6%), oxygenated diterpenes (2.0%), sesquiterpene hydrocarbons (1.1%), and monoterpene hydrocarbons (0.3%). Diterpene hydrocarbons were absent. The major constituents were (*E*)-nerolidol (16.1%), 1,8-cineole (7.3%), *iso*-longifolol (5.8%), and isoamyl dodecanoate (4.3%). Other notable compounds (>1.0%) included *α*-terpineol (3.6%), benzeneacetaldehyde (3.3%), 2-methyl-1,3-cyclohexadiene (3.0%), borneol (3.0%), *n*-octanol (2.3%), (3*Z*)-hexenol (2.0%), benzaldehyde (2.0%), camphor (2.0%), *cis*-phytol (2.0%), *n*-tetradecanol (1.9%), endo-fenchol (1.8%), *n*-nonanol (1.8%), cedroxyde (1.6%), linalool (1.5%), methyl decyl ketone (1.4%), geranyl acetone (1.4%), *n*-nonanal (1.3%), terpinen-4-ol (1.3%), norpatchoulenol (1.3%), bulnesol (1.3%), *n*-Heptadecane (1.3%), fenchone (1.2%), (3*E*)-hexenal (1.1%), (2*E*)-decenal (1.1%), and valerianol (1.0%) (Figure 1).

### 2.2. Biological Activities of EO

#### 2.2.1. Antioxidant and Anti-Tyrosinase Detections

**Figure 2:**
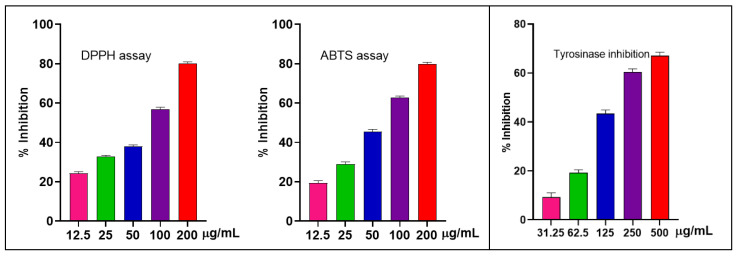
Free radical scavenging activities (DPP and ABTS) and tyrosinase inhibition from *S. japonica* leaf EO.

The antioxidant activity of the EO from *S. japonica* leaves was assessed using 2,2-diphenyl-1-picrylhydrazyl (DPPH) and 2,2′-azino-bis(3-ethylbenzothiazoline-6-sulfonic Acid (ABTS) assays. The EO showed good antioxidant activity with an IC_50_ value of 62.79 ± 0.77 µg/mL for DPPH. A similar trend was observed in the ABTS assay, where the EO demonstrated a good effect with an IC_50_ value of 57.82 ± 1.12 µg/mL. The positive control exhibited lower IC_50_ values of 2.15 ± 0.03 µg/mL for DPPH and 1.95 ± 0.05 µg/mL for ABTS (Figure 2).

Tyrosinase is crucial for melanin formation and is activated under oxidative conditions; thus, samples that efficiently scavenge free radicals may also modulate tyrosinase activity. As a result, the EO had moderate anti-tyrosinase activity with an IC_50_ value of 195.6 ± 3.82 µg/mL compared to vitamin C (IC_50_ ≈ 70.69 ± 1.65 µg/mL) (Figure 2).

#### 2.2.2. The Anti-Inflammatory Effects in LPS-Stimulated RAW 264.7 Macrophages and BSA

The Griess assay was used to determine the inhibitory effects of *S. japonica* leaf EO on LPS-induced NO production in RAW 264.7 cells. EO showed good NO inhibition with the IC_50_ value of 63.03 ± 3.22 µg/mL. To ensure that the observed anti-inflammatory effects were not due to cytotoxicity, cell viability was evaluated in parallel using the MTT assay. Only the adherent cells at the bottom of the culture plate were used to assess viability, reflecting the survival activity of cells following treatment. The results indicated that cell viability remained high, with a survival rate of 95.78% at the highest tested concentration (100 µg/mL). It suggested that the EO is safe for use.

**Figure 3:**
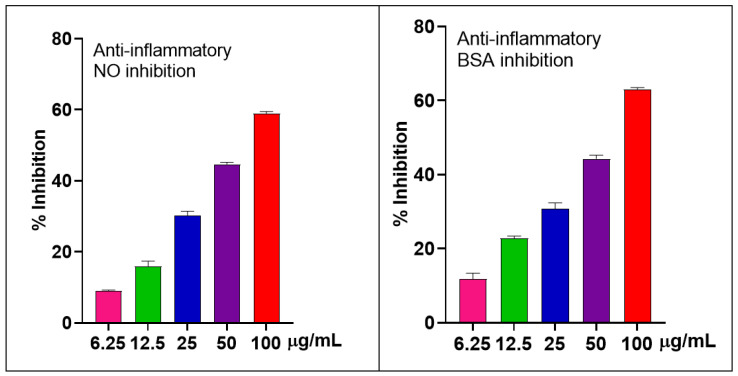
Inhibitions of NO production (%) in LPS-stimulated RAW 264.7 cells and BSA of the *S. japonica* leaf EO.

Regarding BSA inhibition, the pattern was similar to that of NO inhibition. It showed good properties with the IC_50_ value of 59.31 ± 0.71 µg/mL compared to the positive control diclofenac (IC_50_ ≈ 39.19 ± 1.44 µg/mL) (Figure 3).

#### 2.2.3. Anti-α-Glucosidase Activity

**Figure 4:**
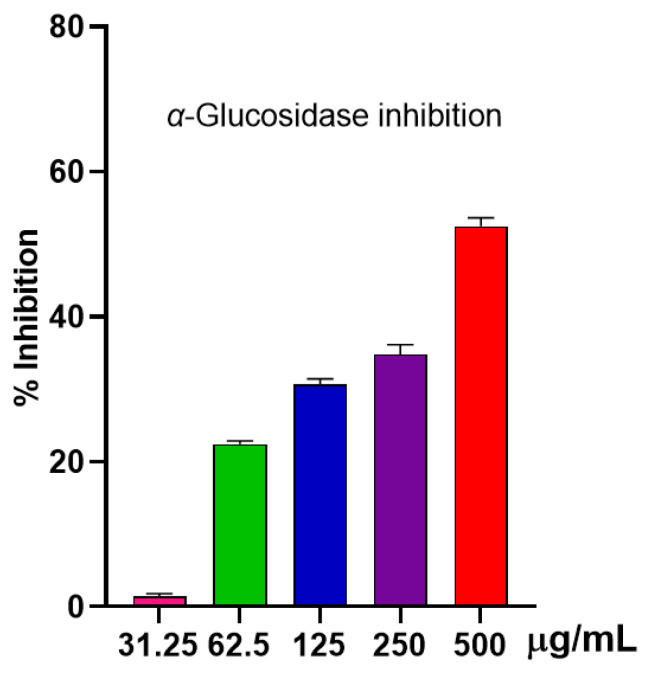
*α*-glucosidase inhibition of the EO.

Shown in Figure 4 is a dose-dependent response to the inhibition of *α*-glucosidase by *S. japonica* leaf EO. A notable inhibition of 52.51 ± 0.91% was observed at 500 µg/mL for the EO, whereas at 250 µg/mL, the inhibition was noticeably decreased at 34.82 ± 1.09%. Although there is some inhibitory capability in *S. japonica* leaf EO, the data suggested that it might not be the best choice for diabetes management.

### 2.3. Molecular Docking Analysis

**Figure 5:**
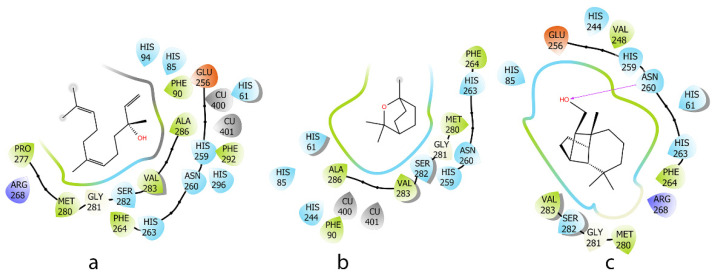
**Two-dimensional** interactions of ligands (*E*)-nerolidol (**a**), 1,8-cineole (**b**), and *iso*-longifolol (**c**) with tyrosinase protein (2Y9X).

The EO showed good antioxidant and significant anti-inflammatory effects. Therefore, the main components such as (*E*)-nerolidol (16.1%) (**a**), 1,8-cineole (7.3%) (**b**), and *iso*-longifolol (5.8%) (**c**) in this EO were evaluated in silico for anti-tyrosinase and anti-inflammatory effects. Tyrosinase is an enzyme involved in melanin production, which can lead to pigmentation issues when overactive. Antioxidants can reduce oxidative stress, inhibit tyrosinase effects, and contribute to anti-inflammatory responses. Regarding the tyrosinase enzyme, a high binding energy indicates a stronger interaction between **a**–**c** and the protein. Three compounds, characterized by a lipophilic carbon framework, showed good energy ranging from −36.3 to −55.7 kcal/mol. Due to their specific chemical structures, the interactions between these three docked compounds and the protein binding site were primarily driven by hydrophobic interactions. Similarly, on the tyrosinase protein, compounds with an OH group (**a** and **c**) showed more favorable interactions with the binding site compared to **b** (Figure 5 and Table 2).

**Figure 6:**
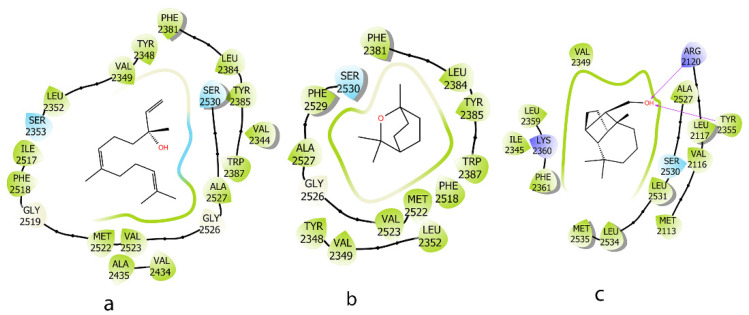
**Two-dimensional** interactions of ligands (*E*)-nerolidol (**a**), 1,8-cineole (**b**), and *iso*-Longifolol (**c**) with COX-2 protein (1CVU).

On the COX-2 protein, compounds **a** and **c**, containing an OH group, demonstrated better MM-GBSA energy compared to **b**, which lacked this functional group. Compound **c** exhibited the strongest binding energy, with its OH group forming two hydrogen bonds with Arg 2120 and Tyr 2355. In contrast, compound **b** engaged only in hydrophobic interactions with the binding site, leading to weaker binding (Figure 6 and Table 3).

## 3. Discussion

### Chemical Profile of EO

The EO of *S. japonica* leaves, non-terpenic compounds predominated at 39.0%, followed by oxygenated sesquiterpenes (31.4%), oxygenated monoterpenes (25.6%), oxygenated diterpenes (2.0%), sesquiterpene hydrocarbons (1.1%), and monoterpene hydrocarbons (0.3%), with diterpene hydrocarbons being absent. Major constituents included (*E*)-nerolidol (16.1%), 1,8-cineole (7.3%), and *iso*-longifolol (5.8%), alongside several compounds exceeding 1.0% abundance. Significant chemotypic divergence is revealed by comparative research within the *Serissa* genus, particularly regarding *S. serissoides*, which shows notable seasonal changes. *δ*-9(10)-tetrahydrocostunolide-1-keto (35.51%), 2-methoxy-4-vinylphenol (10.87%), and 1b,5,5,6a-tetramethyl-octahydro-1-oxa-cyclopropa[a]inden-6-one (7.32%) constitute the majority of the 43 chemicals (78.91% of the total oil) found in *S. serissoides* oil in fall. Germacrene D (12.311%), 5-propionyl-2-chlorobenzeneacetic acid methyl ester (8.541%), and 2-methoxy-4-vinylphenol (6.513%) are the main ingredients of the 72 chemicals that make up the oil in spring (79.88% of the total oil). Interestingly, despite their taxonomic proximity, *S. japonica* and *S. serissoides* do not share any dominating chemicals. In contrast to *S. serissoides*’ mixed terpenic, non-terpenic, and sesquiterpene-rich fall profile, *S. japonica* has a higher predominance of non-terpenic compounds (39.0%), suggesting significant genetic or environmental influence on biosynthesis pathways. Adaptive metabolic plasticity is suggested by seasonal shifts in *S. serissoides* [14].

*S. japonica* is unique among the Rubiaceae family in that it has a higher (*E*)-nerolidol concentration (16.1%) than other species. For example, in *Geophila repens*, *β*-caryophyllene (23.3%), and *β*-elemene (8.0%) predominate, while (*E*)-nerolidol makes up 3.3% [15]. According to earlier research, *S. japonica*’s high concentration of (*E*)-nerolidol indicates increased sesquiterpene production, which may be connected to ecological functions such as herbivore deterrence [15]. Broader comparisons within the Rubiaceae family reveal significant chemical diversity. Spathulenol (10.4%) and thujopsan-2-α-ol (9.5%) are found in *Psychotria laui* oil, whereas (*E*)-citral (20.6%) and 10-epi-*γ*-eudesmol (15.9%) are found in *Psychotria asiatica* oil [16]. Germacrene D (27.70%) is abundant in the flowers of *Galium verum*, while 2-methylbenzaldehyde (26.27%) dominates the leaves. *Cruciata laevipes* is dominated by *cis*-3-hexen-1-ol (9.69%) and *β*-caryophyllene (19.90%) [17]. *S. japonica*’s unique chemotypic profile within the family is further supported by the absence of substantial aldehydes or phenylpropanoids. In summary, *S. japonica* differs from comparable taxa because it has a distinct chemotype within the Rubiaceae family, with a higher (*E*)-nerolidol and a more significant proportion of non-terpenic compounds.

The Rubiaceae family displays antioxidative, anti-inflammatory, and anti-glucosidase effects owing to their prebiotic activity, free radical neutralizing ability, and immune system-modulating characteristics. The biological activity of the EO derived from *S. japonica* leaves was assessed. No published studies have reported the antioxidant, anti-inflammatory, and anti-glucosidase activities of EOs from the *Serissa* genus, rendering this observation a novel addition to the scientific record. Within the Rubiaceae family, comparative insights can be drawn from other species. For example, the EO from *Psychotria asiatica* leaves exhibited anti-inflammatory properties by reducing nitric oxide production, while showing only mild antioxidant activity [16]. This suggests that *S. japonica* EO has a relatively more robust antioxidant profile than *P. asiatica* within the same family. Additionally, the EO from *Coffea arabica* L. husks, another Rubiaceae species, displayed significant in vivo anti-glucosidase and antioxidant effects in a dose-dependent manner in the DPPH assay [18], further illustrating the variability of antioxidant potential within the family. Moreover, the extract of *Psychotria malayana* demonstrated significant potential in managing diabetes and inflammatory responses [19].

In this study, the EO was primarily noted for its antioxidant and anti-inflammatory activities, with the chemical composition consisting mainly of three key compounds: (*E*)-nerolidol (16.1%) (**a**), 1,8-cineole (or eucalyptol) (7.3%) (**b**), and *iso*-longifolol (5.8%) (**c**), along with several minor constituents. The high relative abundance of these compounds, as determined by GC-MS analysis, suggests that they are the dominant bioactive constituents in the EO. There is supporting evidence from the literature that these specific constituents have been previously reported to exhibit antioxidant and anti-inflammatory effects [20,21,22,23]. Therefore, we conducted an in silico analysis using the crystallographic structure of the COX-2 and tyrosinase enzymes. The presence of the hydroxyl group significantly improves the binding affinity of **a** and **c** by facilitating hydrogen bond formation with critical amino acids in the COX-2 and tyrosinase binding site [22,24]. Compound **c**, with its ability to form two hydrogen bonds with COX2, demonstrated the most potent binding, followed by one hydrogen bond with tyrosinase protein. Compound **a** accounted for the highest ratio (16.1%). It relied solely on hydrophobic interactions and showed the second-strongest binding affinity, underscoring the importance of functional groups in achieving strong protein–ligand interactions. This is consistent with prior research identifying (*E*)-nerolidol as a molecule with strong antioxidant and anti-inflammatory effects [20,21]. Similarly, 1,8-cineole, despite having a slightly lower binding affinity, showed considerable interaction with the COX-2 active site, possibly explaining its contribution to the EO’s anti-inflammatory properties [23]. Regarding the inhibition of *α*-glucosidase, there is currently no proof that the primary components of EO have any noticeable inhibitory effects. Our findings indicate that the EO exhibited only weak anti-glucosidase activity, reinforcing that these specific compounds may not play a crucial role in α-glucosidase inhibition. This suggests that the EO is unlikely to contribute significantly to regulating blood glucose levels.

## 4. Materials and Methods

### 4.1. General Procedures

The bioactivity measurements were conducted using a Thermo Fisher Scientific UV-Vis spectrophotometer for cuvette (U.S.) and SH-1200 microplate reader (Corona electric Co., Ltd., Tokyo, Japan) for 96-wells. The positive controls and reagents were obtained from Sigma Aldrich (St. Louis, MO, USA). Dulbecco’s Modified Eagle’s Medium (DMEM) and fetal bovine serum (FBS) were sourced from Waco (Pure Chemical Industrial, Ltd., Osaka, Japan). The RAW 264.7 cells were supplied by the Riken Cell Bank (Tsukuba, Japan). Pure Chemical Industries, Ltd.: Osaka, Japan

### 4.2. Plant Materials

Fresh leaves of Serissa japonica (1.2 kg) were collected in February 2025 from Thua Thien Hue province, Vietnam (Latitude: 16°48′34.8″ N and Longitude: 107°58′88.6″ E). Identification was verified by Thao Xuan Hoang (Faculty of Biology, University of Education, Hue University), and a voucher specimen (SJL-202502) was deposited at the Faculty of Chemistry, University of Education, Hue University.

### 4.3. Hydro-Distillation of EO

The leaves were thoroughly washed, chopped into small fragments, and subjected to hydro-distillation for 4.5 h using a Clevenger-type apparatus, following Vietnamese Pharmacopeia guidelines [25]. The hydro-distillation of 1.2 kg of fresh leaves yielded 1.2 g of EO (0.1% *w*/*w*, fresh weight). The resulting EO was dried over anhydrous Na_2_SO_4_ and stored at 5 °C until analysis.

### 4.4. The GC-MS Analysis

GC-MS analysis was performed on a Shimadzu GCMS-QP2010 Plus system (Shimadzu, Kyoto, Japan) equipped with an Equity-5 fused silica capillary column (30 m × 0.25 mm, 0.25 μm film thickness; Supelco, PA, USA) [26,27]. Helium was used as the carrier gas at a flow rate of 1.2 mL/min. Injector and interface temperatures were maintained at 280 °C. The oven was set to 60 °C (held for 2 min), increased to 240 °C at 3 °C/min (held for 10 min), then raised to 280 °C at 5 °C/min (held for 35 min). Samples (1.0 µL) were injected in split mode (30:1) at an inlet pressure of 93.2 kPa. Mass spectrometry conditions included an ionization voltage of 70 eV, a detector voltage of 0.80 kV, and a scan range of 40–500 amu at 0.5 scan/s. Retention indices (RIs) were calculated relative to a C_7_–C_40_ n-alkane series co-injected with the sample. Compounds were identified by comparing RI values with the literature data and mass spectra with NIST 11 and WILEY 7 libraries [28].

### 4.5. Biological Activities

DPPH, ABTS radical scavenging, and tyrosinase inhibition activities: The antioxidant capacity of *S. japonica* leaf EO to neutralize free radicals produced from DPPH and ABTS, as well as its anti-tyrosinase effect, was assessed using the specified methodology, with requisite adaptations to accommodate the laboratory. The detailed protocols for these assessments have been previously outlined in our work [1,29].

Anti-inflammatory evaluation: The inhibitory effect of *S. japonica* leaf EO on LPS-induced NO generation in RAW 264.7 cells was assessed. The nitrite concentration, was evaluated utilizing the Griess reaction. The cytotoxicity assay was evaluated by MTT assay. Our previous studies have delineated comprehensive procedures for these assays [29].

Bovine serum albumin (BSA) assay: The ability of the EO to prevent the denaturation of BSA was assessed using a modified version described by Sakat et al. (2010) [30]. 10 mg/mL of EO was dissolved in DMSO, and then further diluted in phosphate-buffered saline (pH 6.3) to achieve final concentrations ranging from 6.25 to 100 µg/mL. 500 µL of the sample was mixed with 180 µL of a 5% BSA solution and buffer to make 2 mL. The mixtures were thoroughly mixed and incubated at 37 °C for 10 min, followed by heating at 100 °C for 3 min. After cooling, the absorbance was measured at 660 nm using a UV-Visible spectrophotometer. Diclofenac was used as a positive control [29].

Anti-α-glucosidase assay: The α-glucosidase inhibition experiment was conducted using previously established conditions with minor adjustments. The α-glucosidase enzyme was diluted with 0.1 M sodium phosphate buffer (pH 6.8) to achieve a concentration of 1 U/mL. Samples (500, 250, 125, 62.5, and 31.125 µg/mL) were combined with α-glucosidase solution (20 μL) in a 96-well plate. Then, p-NPG (0.53 mM; 380 μL), the substrate, was introduced, and the mixture was incubated in dry baths at 37 °C for 40 min. The reaction was halted by adding 500 μL of 0.1 M Na_2_CO_3_ solution. The absorbance of the generated *p*-nitrophenol was quantified by assessing the reduction in absorbance at 400 nm [31].

### 4.6. In Silico Analysis

The COX-2 protein (PDB: 1CVU) [32] and the Tyrosinase protein (PDB: 2Y9X) [33] utilized in this investigation were sourced from the Protein Data Bank and processed via the Protein Preparation Workflow [34]. The ligands were prepared using LigPrep. Docking was later performed with Induced Fit Docking with Enhanced Precision [35]. The protein–ligand complex was subjected to energy computation by MM-GBSA to assess the binding affinity and stability of the interactions [36].

Statistical Analysis: The data of bioassays were analyzed using GraphPad Prism, version 9.3.0, GraphPad Software, San Diego, California, with mean ± standard deviation (SD), n = 3.

## 5. Conclusions

For the first time, we reported on both the chemical constituents and biological effects of EO obtained from *S. japonica* leaves. We detected 64 chemical components using GC-MS, with 1,8-cineole, (*E*)-nerolidol, and iso-longifolol accounting for 7.3%, 16.1%, and 5.8% of the oil, respectively. EO inhibited good antioxidants, as evidenced by ABTS and DPPH assays, and exhibited anti-inflammatory activities without cytotoxicity through NO inhibition and BSA denaturation assays. However, it exhibited weak *α*-glucosidase inhibition. *In silico* analysis of the main components of tyrosinase and COX-2 enzymes was conducted to support these activities. Taken together, the *S. japonica* leaves EO was not a strong candidate for diabetes treatment, but its anti-inflammatory without cytotoxicity and antioxidant properties suggested the potential for medicinal applications.

## Figures and Tables

**Table 1:** Chemical composition of *Serissa japonica* leaf EO.

No	Compound ^a^	RT	RI ^b^	RI ^c^	Concentrations (%)	Classification
1	2-methyl-1,3-cyclohexadiene	3.08	767	760	3.0	NT
2	1-octene	3.59	790	788	0.6	NT
3	(3*E*)-hexenal	3.80	799	792	1.1	NT
4	Furfural	4.45	828	828	0.5	NT
5	(3*Z*)-hexenol	4.95	850	850	2.0	NT
6	*n*-hexanol	5.25	863	863	0.6	NT
7	Isopentyl acetate	5.37	868	869	0.3	NT
8	*n*-Heptanal	6.09	900	901	0.4	NT
9	Camphene	7.63	946	946	0.3	MH
10	(2*E*)-heptenal	7.86	953	947	0.4	NT
11	Benzaldehyde	8.01	957	952	2.0	NT
12	*n*-heptanol	8.34	967	959	0.8	NT
13	6-methyl-5-hepten-2-one	8.94	985	981	0.5	OM
14	*n*-Octanal	9.53	1002	998	0.2	NT
**15**	**1,8-Cineole**	**10.69**	**1030**	**1026**	**7.3**	**OM**
16	Benzeneacetaldehyde	11.16	1041	1036	3.3	NT
17	*n*-octanol	12.33	1069	1063	2.3	NT
18	Fenchone	13.09	1088	1083	1.2	OM
19	Linalool	13.56	1099	1095	1.5	OM
20	*n*-nonanal	13.75	1103	1100	1.3	NT
21	*endo*-fenchol	14.18	1113	1114	1.8	OM
22	*cis*-limonene oxide	15.15	1135	1132	0.4	OM
23	*trans*-limonene oxide	15.32	1138	1137	0.6	OM
24	Camphor	15.56	1144	1141	2.0	OM
25	(2*E*)-nonen-1-al	16.22	1159	1157	0.7	NT
26	Borneol	16.52	1165	1165	3.0	OM
27	*n*-nonanol	16.77	1171	1165	1.8	NT
28	*cis*-pinocamphone	16.90	1174	1172	0.6	OM
29	Terpinen-4-ol	17.03	1177	1174	1.3	OM
30	*α*-terpineol	17.64	1190	1186	3.6	OM
31	Methyl salicylate	17.77	1194	1190	0.9	NT
32	*n*-decanal	18.28	1205	1201	0.6	NT
33	*β*-cyclocitral	18.97	1221	1217	0.7	OM
34	Geraniol	20.50	1255	1249	0.2	OM
35	(2*E*)-decenal	20.78	1261	1260	1.1	NT
36	*n*-decanol	21.27	1272	1266	0.6	NT
37	*ρ*-vinyl-guaiacol	23.08	1313	1309	0.8	NT
38	(2*E*,4*E*)-decadienal	23.21	1316	1315	0.9	NT
39	(2*E*)-undecenal	25.26	1364	1357	0.5	NT
40	(*E*)-*β*-damascenone	26.19	1385	1383	0.5	OS
41	Methyl decyl ketone	26.35	1389	1388	1.4	NT
42	(*E*)-Jasmonyl acetate	27.04	1405	1398	0.4	OS
43	Geranyl acetone	29.02	1453	1453	1.4	OM
44	(*E*)-*β*-ionone	30.43	1487	1487	0.7	OS
45	*α*-muurolene	31.02	1502	1500	0.4	SH
46	*δ*-cadinene	31.94	1525	1522	0.7	SH
47	Norpatchoulenol	33.10	1555	1553	1.3	OS
**48**	**(*E*)-nerolidol**	**33.49**	**1565**	**1561**	**16.1**	**OS**
49	*trans*-sesquisabinene hydrate	34.09	1580	1577	0.8	OS
50	*n*-Hexadecane	34.96	1602	1600	0.8	NT
51	Tetradecanal	35.34	1612	1611	0.8	NT
52	1-*epi*-cubenol	36.03	1631	1627	0.7	OS
53	Valerianol	37.02	1657	1656	1.0	OS
54	Citronellyl tiglate	37.48	1670	1666	0.4	OS
55	Bulnesol	37.64	1674	1670	1.3	OS
56	*n*-Tetradecanol	37.76	1677	1671	1.9	NT
57	*n*-Heptadecane	38.58	1700	1700	1.3	NT
58	Cedroxyde	39.12	1714	1713	1.6	OS
**59**	***iso*-longifolol**	**39.76**	**1733**	**1728**	**5.8**	**OS**
60	(*E*)-*β*-santalol	39.93	1738	1738	0.8	OS
61	*n*-pentadecanol	41.41	1779	1773	0.5	NT
62	Isoamyl dodecanoate	43.70	1846	1844	4.3	NT
63	*n*-Heneicosane	51.86	2100	2100	0.8	NT
64	*cis*-phytol	52.27	2114	2113	2.0	OD
Total					99.4	
Monoterpene hydrocarbons (MHs)					0.3	
Oxygenated monoterpenes (OMs)					25.6	
Sesquiterpene hydrocarbons (SHs)					1.1	
Oxygenated sesquiterpenes (OS)					31.4	
Oxygenated diterpenenes (ODs)					2.0	
Non-terpenic compounds (NTs)					39.0	

Concentrations (%) represent the relative proportion of each compound in the total EO (0.1% yield from fresh plant material), determined by GC-MS total ion chromatogram (TIC) peak areas. ^a^ elution order on Equity-5 column; ^b^ retention indices on Equity-5 column; ^c^ literature retention indices (see references); bold: major compounds with ≥5.00%.

**Table 2:** MM_GBSA binding free energy and interaction between ligands and tyrosinase protein (2Y9X).

Compound	Induce Fit Docking XP (Kcal/mol)	MM-GBSA (Kcal/mol)	Interaction
**a**	−4.0	−55.2	-
**b**	−3.3	−36.3	-
**c**	−4.6	−55.7	H-Bond: ASN 260

**Table 3:** MM_GBSA binding free energy and interaction between ligands and COX-2 protein (1CVU).

Compound	Induce Fit Docking XP (Kcal/mol)	MM-GBSA (Kcal/mol)	Interaction
**a**	−6.541	−75.31	-
**b**	−6.457	−55.10	-
**c**	−7.245	−93.96	H-Bond: ARG 2120, TYR 2355

## Data Availability

The original contributions presented in the study are included in the article. Further inquiries can be directed to the corresponding author.
